# Re-Visiting the Incidence of Environmental Factors on a Pre-Imaginal Population of the Red Gum Lerp Psyllid, *Glycaspis brimblecombei* Moore

**DOI:** 10.3390/insects11120860

**Published:** 2020-12-03

**Authors:** Jürgen Junk, Michael Eickermann, Milan Milenovic, Pompeo Suma, Carmelo Rapisarda

**Affiliations:** 1 Environmental Research and Innovation Department (ERIN), Luxembourg Institute of Science and Technology (LIST), 41, Rue du Brill, L-4422 Belvaux, Luxembourg; juergen.junk@list.lu (J.J.); milan.milenovic@list.lu (M.M.); 2 The Department of Agriculture, Food and Environment (Di3A), University of Catania, via Santa Sofia 100, I-95123 Catania, Italy; suma@unict.it (P.S.); carmelo.rapisarda@unict.it (C.R.)

**Keywords:** climatic factors, environmental factors, *Eucalyptus* psyllid, population, sampling methods, Sicily

## Abstract

**Simple Summary:**

The red gum lerp psyllid (*Glycaspis brimblecombei*) is an invasive pest of *Eucalyptus* trees worldwide, responsible for serious damage. A revisited analysis was carried out on data collected in eastern Sicily soon after the psyllid introduction in 2012/13. *G. brimblecombei* has been sampled by two different methods on *Eucalyptus camaldulensis* in nine different sites, where the main climatic data (air temperature, relative humidity, and precipitation) have been registered. *G. brimblecombei* population showed a similar trend in all nine sites, positively correlated only with air temperature. A negative correlation has emerged with precipitation and relative humidity. The results show the need for a deeper understanding of the role played by environmental factors as well as by the sampling methods based on the random collection of a fixed number of leaves, compared to methods based on the collection of infested leaves in a fixed time interval.

**Abstract:**

The red gum lerp psyllid, *Glycaspis brimblecombei* Moore (Hemiptera: Aphalaridae), is an invasive pest of Eucalyptus trees worldwide, responsible for serious damage, including the death of plants. Knowledge about the incidence of climatic factors on the insect development are essential to define useful strategies for controlling this pest. To this aim, *G. brimblecombei* has been sampled by two different methods from April 2012 to February 2013 in eastern Sicily on *Eucalyptus camaldulensis* in nine different sites, where the main climatic data (air temperature, relative humidity, and precipitation) have been also registered. The *Glycaspis brimblecombei* population showed a similar trend in all nine sites, positively correlated only with air temperature, but a negative correlation has emerged with precipitation and relative humidity. The results show the need for a deeper understanding of the role played by other abiotic (such as different concentrations of CO2) and biotic (e.g., the antagonistic action of natural enemies, competition with other pests, etc.) factors. The greater sensitivity, even at low densities of psyllid, of sampling methods based on the random collection of a fixed number of leaves compared to methods based on the collection of infested leaves in a fixed time interval has been also outlined.

## 1. Introduction

The red gum lerp psyllid, *Glycaspis brimblecombei* Moore (Hemiptera: Aphalaridae), is a pest of Eucalyptus trees which has shown invasive behavior worldwide for a couple of decades [1]. Native to Australia, as its host plants are, it was recorded for the first time outside the Australian Region in the USA, in California, in 1998 [2,3]. Since that time, its dangerous presence has been found progressively in many countries of Central and South America, Africa, and Europe. A chronology of reports of *G. brimblecombei* in non-native areas is provided by the literature [4,5]. In Italy, after it was first recorded in the central and southern regions of the country [6], *G. brimblecombei* soon showed a wide diffusion in nearly all *Eucalyptus*-growing areas, including the two main islands, Sardinia and Sicily [7,8,9,10].

In its native region, the psyllid feeds on many species of the genus *Eucalyptus* L’Hér. (Fam. Myrtaceae) [11,12]. Outside the Australian region, *Eucalyptus camaldulensis* Dehnh. represents its main host plant, though it has been reported secondarily also on other species, including *E. diversicolor* F. Muell., *E. globulus* Labill., *E. rudis* Endl., *E. sideroxylon* A. Cunn. ex Woolls, and *E. tereticornis* Sm., with different degrees of susceptibility [13,14,15]. On its host plants, the psyllid almost exclusively attacks the leaves, on which eggs are laid and nymphs develop. Mature nymphs of *G. brimblecombei* create white and almost conical shelters made of special secretions, called “lerp”, under which they complete their development [16,17]. Lerps are easily visible even from a distance on infested leaves and allow the easy detection of this insect on plants.

*Glycaspis brimblecombei* completes 2–4 generations per year in the Australian native region [18], but it may reach up to 6 yearly generations in newly colonized areas [19]. The damage it causes to eucalyptus trees may be serious, consisting of leaf dropping until severe defoliation is achieved, twig desiccation, a decrease in growth rate, reduced timber production, and even the death of infested plants, which have been reported from many countries in South America in the case of outbreaks [20,21]. Moreover, it has been documented how psyllid attacks during the *E. calmadulensis* flowering period can have strong negative impacts on plant health, with consequences on flowers and honey production [22]. The occurrence and diffusion of efficient natural enemies are important factors influencing *G. brimblecombei*’s harmfulness; in addition, various studies that have been performed worldwide have shown how environmental factors (especially air temperature and relative humidity (RH), as well as rainfall frequency or intensity) play an important role in affecting the psyllid development through their influence on parasitoid biology [21,23,24]. In Italy, based on studies carried out in areas where the parasitoid *Psyllaephagus bliteus* Riek (Hymenoptera Encyrtidae) was absent, it has been suggested how the size of the *G. brimblecombei* population in a new colonized area is mainly negatively affected by low winter temperatures, but also by high summer temperatures in the absence of rainfall [25]. More recently, research conducted in Sicily on the distribution and activity of both *G. brimblecombei* and its parasitoid *P. bliteus* has shown the latter species as the main factor negatively influencing the *G. brimblecombei* population; further, it showed how RH (namely, the number of daily hours with an RH >80%) and the average daily maximum temperature are important climatic factors affecting the psyllid’s development [26].

Considering the importance that the red gum lerp psyllid has in the forestry economy and given the need to improve knowledge on the influence of environmental factors on its population, a study was carried out through a revisited analysis of the data collected in eastern Sicily soon after the introduction of this pest (e.g., in its early invasive phase). The results of these analysis are reported in the present paper.

## 2. Materials and Methods

### 2.1. Biological Data Collection

The sampling of *G. brimblecombei* has been realized in *E. camaldulensis* plantations newly infested by the psyllid at nine localities in eastern Sicily (south Italy), located at different latitudes (from 36°48′ to 37°54′ N), longitudes (from 14°27′ to 15°18′ E), and altitudes (from 3 m to 520 m a.s.l.), in the provinces of Messina, Ragusa, and Siracusa (Table 1). In all sites tested, eucalyptus plants were grown naturally, without any artificial intervention relating to fertilization, irrigation, or pest control.

At each site, samplings of the psyllid were carried out from April 2012 to February 2013 at an interval of about 45 days and in different seasons: two in spring (18–28 April and 30 May–6 June 2012), two in summer (12–24 July and 28 August–6 September 2012), two in autumn (11–18 October and 27 November–19 December 2012), and one in winter (1–19 February 2013). For each sampling date, the following two different collection methods, both concentrated in the lower part of the canopy at a maximum height of 2.5 m above the ground level, have been adopted, aimed at estimating the presence of the insect and the extent of its infestation:

(a) a total of 100 leaves were collected randomly from 5 plants (20 leaves/plant), regardless of the presence of specimens of the insect and trying to collect for each plant an average of 5 leaves per cardinal point (this method will be named “*psyllid sampling*” in the following pages);
(b) a 30 min collection of leaves was carried out by two collectors (15 min per each) randomly on all *Eucalyptus* plants of the selected site, regardless of the number of leaves sampled (higher on less infested trees, of course, due to the longer time spent looking on the canopy for leaves bearing lerps) but oriented to pick up only leaves showing mature lerps of the insect (this method will be named as “*infestation sampling*” in the following pages). The number of leaves sampled in this method was not recorded.

All the collected material, kept separate based on the two different sampling techniques, was brought to the laboratory, where the young stages (mobile) and nymphs of *G. brimblecombei* were counted by means of a stereomicroscope. The two collecting methods allowed us to obtain two different kind of values: (a) the total number of young stages (mobile) and nymphs/100 leaves collected randomly, and (b) the total number of young stages (mobile) and nymphs/30 min of collection on infested leaves.

As to the adults, their density showed substantial variation throughout the year, which made it difficult to standardize the collection method, and therefore they have not been considered in this study.

### 2.2. Climatic Data Collection

The service provided by the Sicilian Agrometeorological Information Service (SIAS), of the Department of Agricultural and Food Resources of the Sicilian Region, was used to obtain climatic data registered daily in each survey site, with special reference to the mean daily air temperature (°C), the daily precipitation (mm/day), and the daily mean relative humidity (%).

### 2.3. Data Treatment

Due to the way in which they were collected, the biological data indicate values related to the abundance of *G. brimblecombei* populations in the investigated sites, whatever the sampling methods were. The fact that their relief was based on two different types of units (“substrate unit” in the case of the “*psyllid sampling*”, with a randomized survey on a fixed number of leaves, and “time unit” in the case of the “*infestation sampling*”, with exclusive collection during a fixed time of leaves showing the minimal presence of the insect) allowed us to cross-check the results as well as study the reliability of both methods in monitoring the *G. brimblecombei* population under different climatic and ecological conditions.

In both survey systems, counts have exclusively concerned the living and non-parasitized young stages (mobile) and nymphs of the psyllid. For biological data collected with the method of “*infestation sampling*” (e.g., for 30 min on infested leaves), the original set of data were transformed as *y* = log (1 + *x*/30), with *x* being the total number of specimens collected in 30 min.

Regarding the climatic data, unlike in a previous study [26] that grouped all climatic data referred to the 45 days preceding each sample date, in the present study all the data regarding air temperature, precipitation, and relative humidity during the whole study period were considered and analyzed in their original daily frequency.

**Figure 1:**
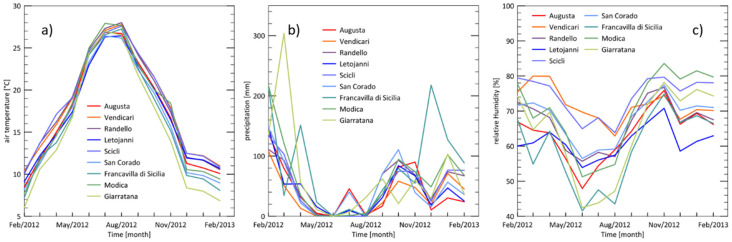
Monthly mean values of air temperature (**a**), total precipitation (**b**), and relative humidity (**c**) in the nine test sites. Time range: February/2012–February/2013.

**Figure 2:**
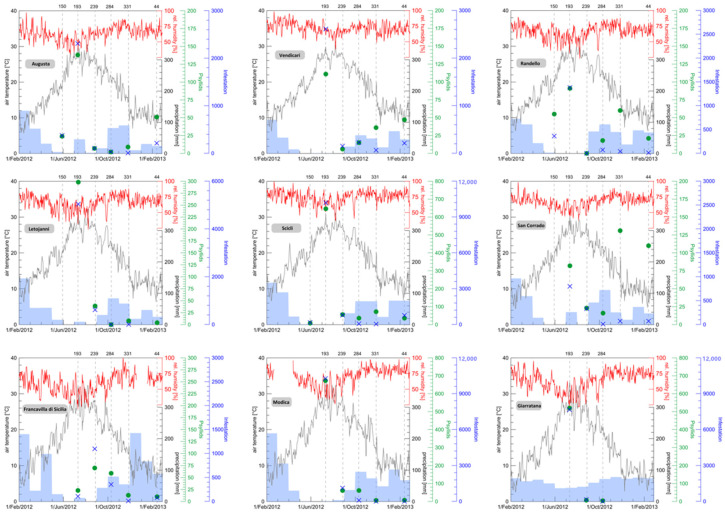
Biological data deriving from the “psyllids sampling” (green dots) and the “infestation sampling” (blue crosses) (given on the secondary axis on the right) correlated to the daily mean values of air temperature and relative humidity and the daily totals of precipitation for the nine test sites. Time range: 01/02/2012–28/02/2013.

Time series of meteorological data were first subjected to an automatic quality control and gap detection method [27]. Less than 1% of gaps were detected in the daily time series of all meteorological data. Gaps of only one day were closed by linear interpolation, with larger ones using corresponding data from the nearest station. Completed time series were used for the plotting in Figure 1 and Figure 2. For the further statistical analysis, the monthly mean values of mean air temperature, the mean relative humidity, as well as the monthly totals of precipitation were calculated. In addition, the cumulative precipitation amount until each date of sampling was calculated. Furthermore, the accumulated temperature sums were computed using the daily mean temperatures with a base temperature of 5 °C as a generally used threshold in the absence of exact data for *G. brimblecombei.*

Rstudio version 1.2.5001 with R version 4.0.2 patched was used in testing for significant difference among different stations as follows. Generalized least squares (GLS) model with within-group correlation structure was separately fitted for temperature, relative humidity and precipitation data with nine stations as factor. To test for pairwise differences between sites, a Tukey HSD post-hoc test was performed using glht function of R “multcomp” package with a significance level of alpha = 0.05. Letter grouping was generated based on p-values. Appropriate correlation structure was selected as follows. To find a correlation structure suitable for data across all sites, automatic structure selection was used as implemented in the auto.arima function of R “forecast” package by applying it to a time series consisting of concatenated data from all nine sites with a sequence of missing values in between separating them. The number of missing values was chosen to ensure the seasonal period was retained, while preventing any serial correlation between the series. In addition, a matrix of external regressors corresponding to nine sites was specified to account for potentially different means between series while keeping other coefficients in common. The selected structures were ARIMA (4,0,1), ARIMA (5,0,0), and ARIMA (5,0,0) for temperature, humidity, and precipitation, respectively. Selected model was confirmed by examining ACF and PACF residual plots and defined in the GLS model function as a correlation structure.

All the other statistical analyses were performed using SigmaStat software Version 10 (Build 10.0.0.54). For the detection of possible correlations between data deriving from the “psyllid sampling”, from the “infestation sampling” and the meteorological variables the Spearman rank-order correlation coefficient as a nonparametric measure of the strength and direction of association between two variables were used. Data were cumulated across sampling sites.

## 3. Results

The main climatic data (air temperature, totals of precipitation, and relative humidity) collected in the nine sites throughout the whole study period are graphically reported in Figure 1.

**Figure 3:**
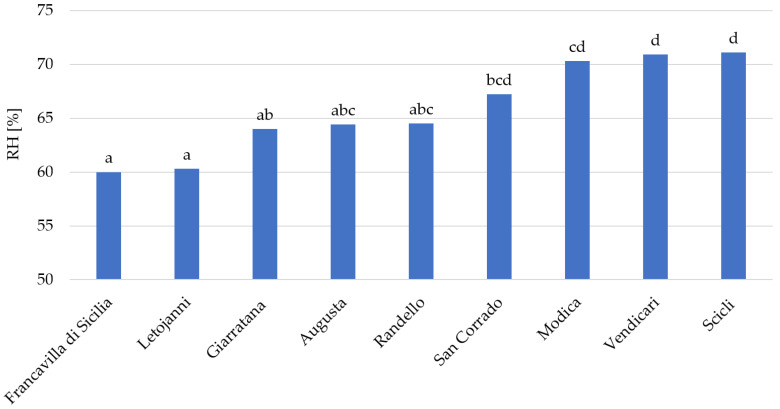
Differences in the mean monthly relative humidity among the nine sites under study. Letters represent significant differences. Sites sharing the same letter are not significantly different.

The statistical analysis showed that there are no significant differences in temperature between the nine sites. Similarly, for precipitation, only one site (Francavilla di Sicilia) showed significantly different and higher precipitation than all sites except Modica and Giarratana. In contrast, there were significant differences in relative humidity between some of the sires, as presented in the Figure 3.

Biological data deriving from both sampling methods (“psyllid sampling” and “infestation sampling”), in relation to the main climatic data (daily mean values of air temperature and relative humidity, and daily totals of precipitation) collected in the nine sites throughout the whole study period, are reported in Figure 2. The range of infestation varied between 0 and 2310 for the psyllid method across the sites, and between 0 and 10,260 for the “infestation sampling” method.

With the exception of some slight differences between the nine sites (with generally higher levels of the presence of the psyllid in the sites of Scicli, Modica, and Giarratana), the population of *G. brimblecombei* showed a similar trend in all the nine sites studied, with peaks highlighted during the summer months, followed by a rapid decrease in the following autumn and, above all, during winter. Both sampling methods agree that Francavilla di Sicilia had the highest infestation levels in August, while, in all other sites, the highest infestation levels were recorded in July sampling. The one exception is San Corrado, where the “psyllid sampling” method produced highest number in November.

The increase in the psyllid population seems to start with considerable earliness, already at the end of winter, in the lower altitude sites.

**Figure 4:**
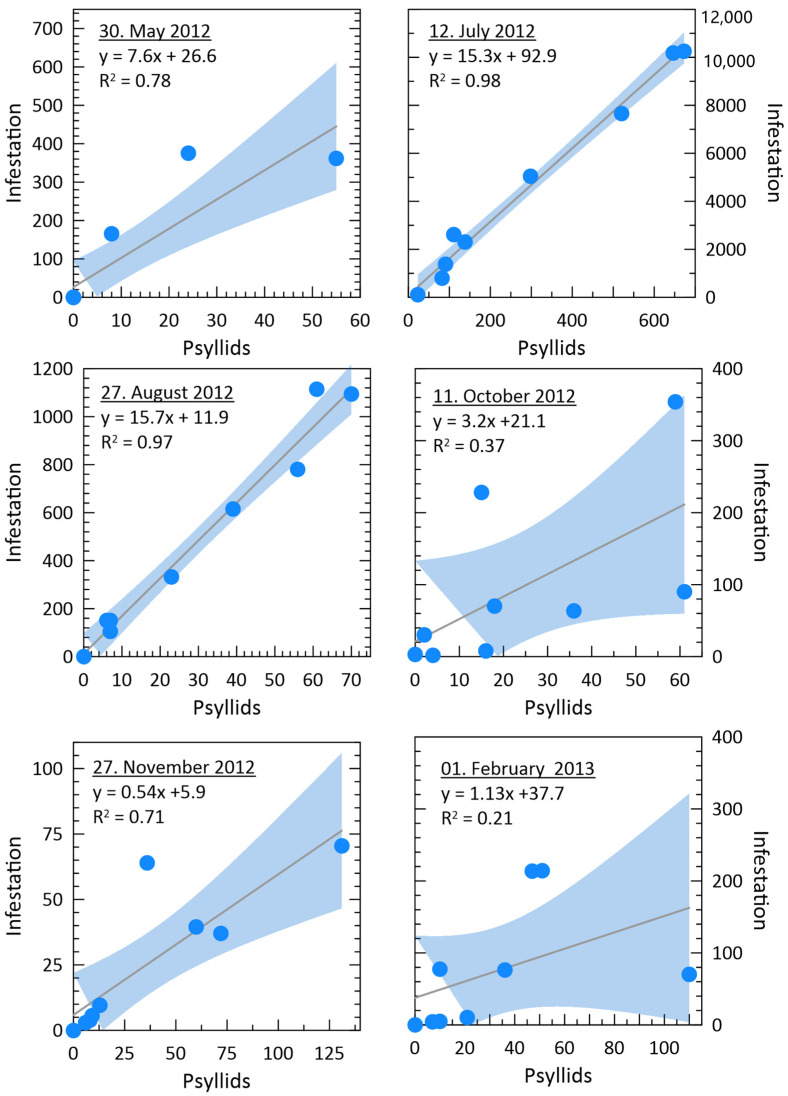
Scatterplots of data coming from psyllid and infestation sampling for all six sampling dates and all test sites (each blue dot represents one test side; blues shaded area (95% confidence interval)).

As for the level of correlation between the two sampling methods used (“psyllid sampling” and “infestation sampling”), this was very high during the summer months (coinciding with the highest population levels of the psyllid), with no relevant differences in the results coming from the two methods, but decreased in the following autumn and winter months, when the “psyllid sampling” showed greater sensitivity under lower population levels of *G. brimblecombei* (Figure 4). Therefore, two methods produce different results when comparing average infestation among sites. The highest contrast is in Augusta, which, according to the “psyllid sampling” method, is the most infested site, while the “infestation sampling” method ranks Augusta as the lowest. Excluding this site, the lowest infestation was the Francavilla di Sicilia site, according to both methods.

The results of the correlation analysis with the Spearman rank-order correlation are summarized in Table 2. During most of the reproductive season (spring and summer), there is a significant correlation between the biological data deriving from “psyllid sampling” and “infestation sampling” methods. Whatever the method used for the collection and evaluation of biological data (“psyllid sampling” or “infestation sampling”), the population of *G. brimblecombei* has a positive and statistically significant correlation only with temperature (both mean and accumulated) and, differently, a negative correlation with both the precipitation (both simple and accumulated) and the relative humidity, However, the statistical significance of this negative correlation is verified only for biological data collected with the “infestation sampling” method.

## 4. Discussion

As for many organisms, ecological models are useful to in predicting the potential spread of *G. brimblecombei* worldwide [21]; they are also important for setting up management and control strategies for the pests. Nevertheless, the transferability of predictive models to changing conditions can be critical and needs to improve knowledge on the determinants of ecological predictability, especially the intrinsic characteristics of the species, biotic interactions, and the quality of data collected [28,29]. Stimulated by the recent rapid spread of this psyllid worldwide, intense research has been developed over the past few years on relationships between climatic factors and the development capacity of *G. brimblecombei*. Reliable data on these relationships can help constructing predictive models. At present, however, the available literature shows data that do not always fully coincide. In our final analysis, it is clear how, among climatic factors, the population level of *G. brimblecombei* is influenced exclusively by air temperature, which however did not show statistically significant differences between the nine sites examined in the present study.

Regarding the effects of temperature, the population size of *G. brimblecombei* in a new area of colonization is reported to be negatively affected by low winter temperatures; the psyllid development increases significantly when the average temperature exceeds 20 °C in central Italy [25]. High summer temperatures, in the absence of rainfall, also have negative effects on the *G. brimblecombei* population, as noted both in Italy [25] and Portugal [30]. The average maximum temperature is reported as the most significant factor positively influencing *G. brimblecombei* infestation [26].

As to the effects of precipitation [31], an experimental test conducted on seedlings of *E. camaldulensis* has proven the effects of artificial rain in reducing the abundance of lerps through their solution and mechanical removal by water. Partly in contrast with this, it was stated that the higher mean values of eggs and nymphs per leaf, with consequent greater infestation levels by the psyllid, derive from the precipitation during periods with mean temperatures exceeding 22 °C [25]. According to this, *G. brimblecombei* may be favored by summer precipitation, or it could benefit from rains after a period of dryness due to their action on physiology, lymphatic circulation, and the degree of hardening of the leaf tissues in the host trees.

In apparent contrast to what has been reported in the literature [26], our results indicate that air temperature (both mean and accumulated) is the main factor influencing *G. brimblecombei* population. No significant relation emerges from our analysis between the psyllid development and both precipitation and humidity. The lack of a statistically significant relationship with the only climatic parameter that showed valid differences between the nine sites studied—i.e., relative humidity—seems to explain the limited differences in the trend of the insect population between the nine sites. Similarly, and in the opposite direction, the lack of significant differences in the population trend of *G. brimblecombei* can be fully correlated with the absence of valid differences in the temperature trend between the sites studied.

Based on the previous considerations, it can be assumed that, though small, the differences highlighted in the psyllid population between the nine sites compared in this study could be attributed to ecological factors unrelated to the examined climatic ones, as already hypothesized in the literature [26]. Among biotic elements, interspecific competition is an important factor affecting the fitness of phytophagous insects [32]. In this context, interesting results have been obtained studying the potential interaction between the two invasive pests of *Eucalyptus* trees: the bronze bug, *Thaumastocoris peregrinus* Carpintero et Dellapè (Heteroptera: Thaumastocoridae) and *G. brimblecombei* [33,34]. Additionally, a deeper understanding of the factors influencing *G. brimblecombei* population may derive from the evaluation of the parasitization activity by *Psyllaephagus bliteus* Riek (Hymenoptera Encyrtidae), which has been shown to be the main factor negatively influencing the *G. brimblecombei* infestation in Italy, causing up to a 64% reduction in the psyllid population [23,26]. Therefore, additional investigations are needed on a wide geographical base to better understand the role of biotic factors and their joint action with abiotic ones.

Furthermore, temperature, precipitation, and relative humidity could not be the only environmental abiotic factors to be considered when studying the climate impact on the *G. brimblecombei* population. The variation in atmospheric CO_2_ concentration, which can significantly modify the nutritional quality of plants, can determine subsequently substantial influences in the growth and development of associated herbivorous insect species. Therefore, clarifying the effects of increasing CO_2_ levels in plant–insect interactions can represent a crucial turning point to cope with, considering that the way populations of herbivorous insects respond to rising CO_2_ levels has not yet been deeply studied [35].

In summary, the results presented here partly confirm various elements reported in the literature but also partly contrast others previous findings. Above all, however, they express the strong need for comprehensive monitoring strategies considering not only climatic parameters but also other environmental abiotic elements, allowing for a deeper and better understanding of the complex non-stationary biotic plant-phytophagous system.

Regarding the sampling methods used in this work, it is worth noting the greater suitability of the “*psyllid sampling*” (based on a random collection of a fixed number of *Eucalyptus* leaves) vs. the “*infestation sampling*” (based on an oriented collection of infested leaves in a fixed time interval) for species monitoring. In fact, during the study reported here, the first sampling method was shown to be more representative of the real infestation status of the psyllid in the field during months characterized by a lower presence of the insect. In order to develop monitoring strategies for *G. brimblecombei* aimed at assessing the adequate integrated control of its infestations, this finding can be of importance, especially considering that, a few years after its invasive introduction in Europe and also thanks to the limiting action performed by its natural enemies that have settled in the meantime, *G. brimblecombei* usually does not show extremely high levels of population anymore in most *Eucalyptus*-growing areas. Monitoring the insect in low to medium population densities has proven to be challenging, and more sensitive monitoring methodological instruments are certainly to be desired and need to be developed in future studies.

## 5. Conclusions

The results from this study, carried out on naturally growing *E. camaldulensis* plants at the stage of initial infestation by *G. brimblecombei*, show how air temperature is the main factor influencing the psyllid population, which apparently has no significant relation with both precipitation and humidity. As temperature did not show statistically significant differences between the nine sites examined in the present study and cannot explain by itself the differences noted during this study in the infestation levels of the psyllid, the latter ones are most probably influenced also by a combination of other abiotic (e.g., variation in atmospheric CO_2_ concentration) and biotic (e.g., interspecific competition with natural enemies and/or other pests) factors.

Regarding the sampling methods tested in this work, aiming at defining their reliability for monitoring the psyllid population at different densities and environmental conditions, the psyllid sampling based on a random collection of a fixed number of *Eucalyptus* leaves provided more reliable results throughout the year and even at low infestation levels.

## Figures and Tables

**Table 1:** East Sicilian sites where biological data on *Glycaspis brimblecombei* and climatic data were collected (reported in ascending order of elevation).

No.	Locality	Province	Altitude (m a.s.l.)	Latitude	Longitude
1	Augusta	Siracusa	3	37°14′36.35″ N	15°12′16.20″ E
2	Vendicari (Noto)	Siracusa	5	36°48′15.70″ N	15°05′34.10″ E
3	Randello (Ragusa)	Ragusa	15	36°50′25.00″ N	14°27′38.80″ E
4	Letojanni	Messina	30	37°52′55.25″ N	15°18′09.55″ E
5	Scicli	Ragusa	200	36°48′45.00″ N	14°42′57.00″ E
6	San Corrado (Noto)	Siracusa	320	36°55′55.00″ N	15°03′32.90″ E
7	Francavilla di Sicilia	Messina	425	37°54′36.65″ N	15°07′42.60″ E
8	Modica	Ragusa	460	36°52′38.70″ N	14°45′12.70″ E
9	Giarratana	Ragusa	520	37°02′53.50″ N	14°47′58.00″ E

**Table 2:** Correlation matrix of the meteorological variables and biological data deriving from “psyllid sampling” and “infestation sampling”, based on monthly groupings. Correlation coefficients and the *p* values (in brackets) are given (significant pairs = grey shaded). For pairs with *p* values greater than 0.05, there is no significant relationship between the two variables; *N* = 54.

Parameters	Precipitation	Humidity	Accumulated Temperature	Accumulated Precipitation	Psyllids	Infestation
Temperature	−0.49 (0.00)	−0.52 (0.00)	0.24 (0.08)	−0.24 (0.08)	0.60 0.00	0.75 0.00
Precipitation		0.55 (0.00)	0.37 (0.01)	0.46 (0.00)	−0.10 (0.45)	−0.29 (0.04)
Humidity			0.41 (0.00)	0.15 (0.27)	−0.19 (0.17)	−0.40 (0.00)
Accumulated Temperature				0.40 (0.00)	0.42 (0.00)	0.29 (0.03)
Accumulated Precipitation					0.08 (0.56)	−0.07 (0.64)
Psyllids						0.91 (0.00)
